# Sustainability potentials of novel laccase tinctures from *Stenotrophomonas maltophilia* BIJ16 and *Bordetella bronchiseptica* HSO16: From dye decolourization to denim bioscouring

**DOI:** 10.1016/j.btre.2019.e00409

**Published:** 2019-12-03

**Authors:** John O. Unuofin

**Affiliations:** a SA-MRC Microbial Water Quality Monitoring Centre, University of Fort Hare, Private Bag X1314, Alice, 5700, South Africa; b Applied and Environmental Microbiology Research Group (AEMREG), Department of Biochemistry and Microbiology, University of Fort Hare, Private Bag X1314, Alice, 5700, South Africa; c Department of Environmental, Water and Earth Sciences, Tshwane University of Technology, Private Bag X680, Pretoria 0001, South Africa

**Keywords:** Environmental sustainability, Dye decolourization, Denim bleaching, Laccase

## Abstract

The aim of this present study was to investigate the environmental proficiency of two laccase producing bacterial strains, Hb16c and Berl1^1^b_2_. Here, laccases, which were secreted in media containing environmental wastes, were characterized for biochemical and kinetic novelty and applied in the decolourization of some synthetic dyes and subsequently, denim bleaching. The laccases exhibited enhanced pH-, thermo-, psychro-, metal-, halo-, and surfacto-tolerance, eliciting residual activities of at least ca. 71%. Thereafter, the enzymes were able to decolourize novel high concentrations of synthetic dyes (0.2% w v^−1^) at 56 h of incubation, and also elicit a mediator-assisted perpetual wash up and decolourization of indigo pigment from fabric under 6 h. The outcomes observed in this study therefore warrant the adoption of these isolates for applications toward a sustainable and total environment through production of fine biochemicals, and the minimization of environmental wastes.

## Highlights

• Laccase from Hb16c and Berl1^1^b_2_ exhibited remarkable polyextremotolerant properties.
• A novel high concentration of synthetic dyes was efficiently degraded.
• Bioscouring of denim was achieved by a mediator-associated laccase treatment.
• Multiple laccase-encoding genes were observed in a laccase-producing strain.

## 1 Introduction

According to the United Nations (UN), under the United Nations Development Programme (UNDP), the sustainable development goals (SDG) or Global Goals, which were inaugurated in January 2016, were intended for the alleviation or extermination of overarching global challenges, such as: poverty, climate and environmental degradation, inequality, injustice, feuds and hostilities, worldwide. The anticipated deliverables of major stakeholders set out in the SDGs by 2030 has engendered the partnership of government, private and different research sectors to collectively pursue a sustainable society. One out of several issues of growing concern is the need for a sustainable environment, which is pivotal to health and socioeconomic development. Humankind has been burdened with the enigma of providing a green environment; especially clean safe water, for a considerable fraction of the world’s population. Moreover, with the continuous depletion and insufficiency of freshwater sources, which are being contended for by the increasing human population and industrialization, the reuse of wastewater is becoming sine qua non for alleviating the stress conferred on available water resources. Interestingly, there is growing concern on how anthropogenic activities have accelerated the overshoot in water pollution, especially through the discharge of high volumes of untreated or inadequately treated wastewater. Amongst them, the manufacture of indigo coloured denims with a stone washed appearance, through conventional physicochemical techniques, is consistently adding to the environmental footprints of denim processing, since they entail the expenditure of phenomenal volumes of water, synthetic dyes, salts, ions, hazardous chemicals and other particles [1]. Therefore, the wastewater from the denim industry could be perceived as a conglomerate of outfalls from different processing units, and more toxic than their effluents, individually, since it might contain chief pollutants from several other batch effluents. The unfavourable environmental feedback of the techniques famously applied, so far, has necessitated the exploration of environmentally friendly modes of denim processing. The use of laccase is being given focal consideration for denim bioscouring due to its ability to catalyze the inexpensive oxidative degradation of a wide array of phenolic and non-phenolic chemicals, as well as inorganic pollutants, thereby generating infinitesimal waste quotients, post-treatment (Unuofin et al. unpublished). Moreover, the espousal of bespoke laccase-based products for denim bleaching by enzymes companies, such as Novozyme (Denmark), Prozyme® LAC (China) Hypozyme (USA), who have constantly produced fungal laccases, and MetGen (Finland), who launched the first commercial bacterial laccase, Metzyme, has motivated for the heuristic search for sustainable sources of laccase production, and the sustainability evaluation of laccase application. In this regard, this study aimed at presenting the life cycle assessment of some bacterial laccases, which had been produced from some environmental wastes [2,3], in the decolourization of some synthetic dyes and the bleaching of indigo stained denims. Especial focus was devoted to the novel characteristics exhibited by these secretions, and a molecular snapshot of multi-homologous laccase coding genes was inferred, just we had discovered in a previous study [4].

## 2 Materials and methods

### 2.1 The isolate

Laccase-producing strains Berl1^1^b_2_ and Hb16c had been isolated from a WWTP influent [4] and a moist decaying wood litter (Unuofin et al. unpublished), and identified as *Achromobacter xylosoxidans* HWN16 and *Citrobacter freundii* LLJ16, with accession numbers MF073257 and MF073260 respectively from GenBank, National Center for Biotechnology Information (NCBI). They were retrieved from the Biocatalysis Chest in the Applied and Environmental Microbiology Research Group (AEMREG) culture collection. Axenic cultures of Berl1^1^b_2_ and Hb16c were inoculated in agroindustrial wastes-supplemented basal media, which had been optimized in previous investigations [3,4]. The shake flasks containing the optimized media were incubated in an orbital shaker at 30 °C for 120 h, at the respective agitation speeds recommended in previous studies [3,4]. Aliquots of crude extracts were harvested post-incubation and centrifuged at 15,000 rpm for 12 min at 4 °C using a benchtop centrifuge (SIGMA-1-14k). The supernatants were used to assay for protein concentration and laccase activity, respectively.

### 2.2 Protein estimation and laccase activity assay

The Folin phenol reagent was adopted in determining the protein concentration, with a method that coincided with Lowry et al. [5], using bovine serum albumin as standard. Laccase activity was estimated as the catalysis of 2,2’-azino-bis (3-ethylbenzothiazoline-6-sulfonic acid) (ABTS), on observation [6]. A 50 μL aliquot of appropriately diluted crude laccase was reacted with the substrate, ABTS, in potassium phosphate buffer (pH6) at 30 °C, and the reaction was terminated after 10 min with 40 μL 20% Trichloroacetic acid (TCA). Oxidation of ABTS was monitored spectrophotometrically at 420 nm (ε = 36,000 M^−1^ cm^−1^) using a SynergyMX 96-well microtitre plate reader (BioTeK Instruments). One unit of enzyme activity was deciphered as the amount of enzyme oxidizing 1 μmol of ABTS per minute under the aforementioned conditions.

### 2.3 Assay of biochemical novelty of the laccases

The pH optima of the laccases were evaluated at 30 °C for 30 min. in buffers with pH values ranging from 3 to 11. Conversely, the temperature optima were assessed at temperatures ranging from 0 to 90 °C in phosphate buffer (pH 6) for 30 min.. Regarding stability studies, crude laccases were incubated in different buffers (pH 3–11) over a period of 455 min., whereas temperature stabilities for 40−90 °C were measured over 1440 min.. Furthermore, the effect of selected metal ions (Cu^2+^, Zn^2+^, Mg^2+^, Co^2+^) and surfactants (EDTA, benzoic acid and SDS) were assayed after 30 min. of preincubation with crude laccases at the concentrations 1 mM, 2.5 mM, 5 mM and 7 mM, respectively. Solvents (DMSO and Tween 20) and NaCl were assayed for at concentrations of 5%, 10%, 20%, and 40%, respectively. However, NaF, which reportedly has strong inhibitory activity on laccases was evaluated at 2%, 5%, 10%, and 20% concentrations, respectively. The assessment of the aforementioned parameters were conducted, using ABTS as assay substrate. Furthermore, substrate specificity studies were conducted using the following substrates: ABTS, guaiacol, 1-napththol, 2,6 - Dimethoxyphenol (DMP), potassium ferrocyanoferate (PFC), pyrogallol, and syringaldazine, while the kinetic values were determined for the best substrate. Assays were conducted in triplicates.

### 2.4 Detection of laccase-encoding genes

Total nucleic acid of the bacterial strains harvested from nutrient broth by centrifugation was extracted according to the method of Queipo-Ortuno et al. [7], with modifications. The previously washed cells were resuspended in molecular grade nuclease free water, twice after spinning at 15,000 × g for 10 min, and were boiled in an AccuBlock Digital dry bath (TECHNE, Lasec, SA) at 100 °C for 10 min.. Thereafter, the lysates were quickly but briefly cooled on ice, and then spun at 13,500 × g for 5 min. to separate the cellular debris from the clear supernatant containing the total nucleic acid. The clear supernant was aseptically decanted and stored at −20 °C. Aliquots of 5 μL of template DNA were thereafter used for PCR. The primers used, reaction volumes, and cycling conditions were sourced from our previous investigation [4], and were run on a thermal cycler (G-STORM, UK). The amplicons were run on 1.7% agarose gel (Merck, SA) in a Submarine Electrophoresis System (Mupid-One, Takara, ADVANCE Co., Ltd. Japan) for 45 min. at 100 V, and thereafter had their bands visualized with the aid of ethidium bromide stains (Sigma-Aldrich, South Africa) in a transilluminator (ALLIANCE 4.7, France) with the UVITEC Cambridge software.

### 2.5 Dye decolorization

The laccases were assessed for decolorizaton of synthetic dyes: Azure B (AB), Malachite Green (MG), Reactive Blue (RB), Methyl Orange (MO), Congo Red (CR), and Brilliant Blue (BB). A 50 mL homogenate of individual synthetic dyes (100 mg) was prepared using phosphate buffer (50 mM, pH 6). Their maximum absorbance and chemical structures are appended in Table 1. Control treatments contained the decolourization mixtures without the crude laccases. Two milliliters (2 mL) of reaction mixture comprised 50 μL of crude laccase and 1950 μL dye solution. All the decolourizing reactions were carried out at 30 °C and absorbance lectures were taken intermittently for over 80 h with the aid of a Synergy MX Microplate reader (BioTek™) at the respective wavelengths. Thereafter, their decolorization efficiencies were calculated as follows:

% decolourization = (*A*_initial_−*A*_observed_ / *A*_initial_) × 100

Where, *A*_initial_ is the initial absorbance, and *A*_observed_ is the observed absorbance.

**Table 1:** Chemical structure and maximum absorbance of different classes of dyes.

Dye	Type	Wavelength of maximum absorbance (nm)	Chemical structure
Azure b (AB)	Heterocyclic cationic	651	
Reactive Blue (RB)	Anthraquinone	595	
Methyl Orange (MO)	Azo	464	
Congo Red (CR)	Azo	490	
Malachite Green (MG)	Triarylmethane	617	
Brilliant Blue (BB)	Triarylmethane	590	

### 2.6 Denim bioscouring

A parallel experiment was conducted, where a denim garment purchased from a retail shop in Alice, South Africa was trimmed into square portions, and were thereafter exposed to two treatments viz; (i) crude laccase and (ii) crude laccase +2 mM ABTS. However, control bottles were provided which showed the treatment of 2 mM ABTS only. The bottles were incubated at 30 °C, 120 rpm for a period of 6 h. Thereafter, each square portion was gently rinsed with running water to prevent abrasion of fabric or influence dye wash-down. The denim pieces were air-dried and observed under the dissection microscope (× 30) (KYOWA TOKYO No.751252). Their absorbance lectures were afterward taken at 200 nm–900 nm wavelength range with a spectrophotometer (UV-3000 PC), which were later converted into reflectance values by the derived equation:

(1) R = 10^−A^

Where R = reflectance, and A = absorbance value.

### 2.7 Data analysis

Results of replicates were pooled and expressed as mean ± standard deviation (SD) using Microsoft Excel Spreadsheet. Data were subsequently subjected to one-way analysis of variance (ANOVA) and the least significant difference was carried out. Significance was identified at *P ≤ 0.05*.

## 3 Results and discussion

The increasing awareness of the adverse effects of environmental pollution, and the desperate need for a green community, worldwide, has warranted the espousal of microbial technologies toward the initiation and maintenance of environmental sustainability. In consequence, the sustainability annals of the present study had begun from preceding studies, which had expounded the isolation of some remarkable laccase producing bacteria from some environmental wastes and detritus in milieus proposed as ideal mesocosms [4; Unuofin et al. unpublished], and the subsequent valorization of inexpensive agroindustrial wastes as feedstock for the tremendous outputs of laccase, using the auspicious bacterial strains that had been identified [2,3]. Conversely, this study ultimately focuses on the evaluation of the aforesaid biomolecule for suitability in environmental sustainability.

### 3.1 Biochemical novelty of the laccases

**Fig. 1:**
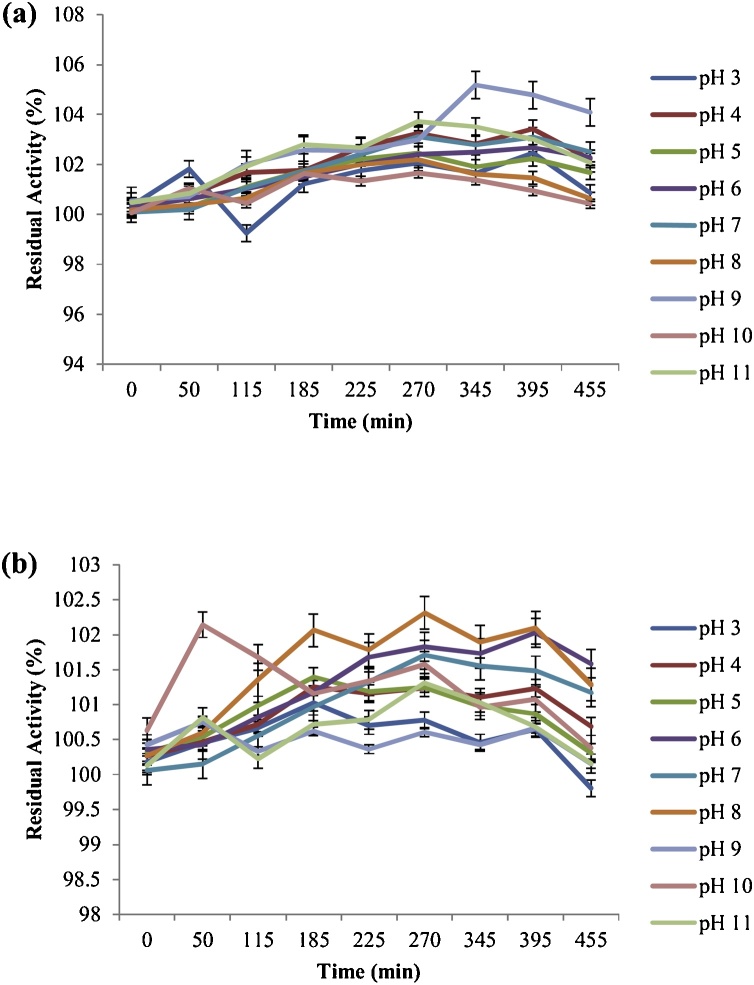
Effect on pH on the stability of laccases from (a) Hb16c (*Bordetella bronchiseptica* HSO16) and (b) Berl1^1^b_2_ (*Stenotrophomonas maltophilia* BIJ16) with ABTS as substrate.

**Fig. 2:**
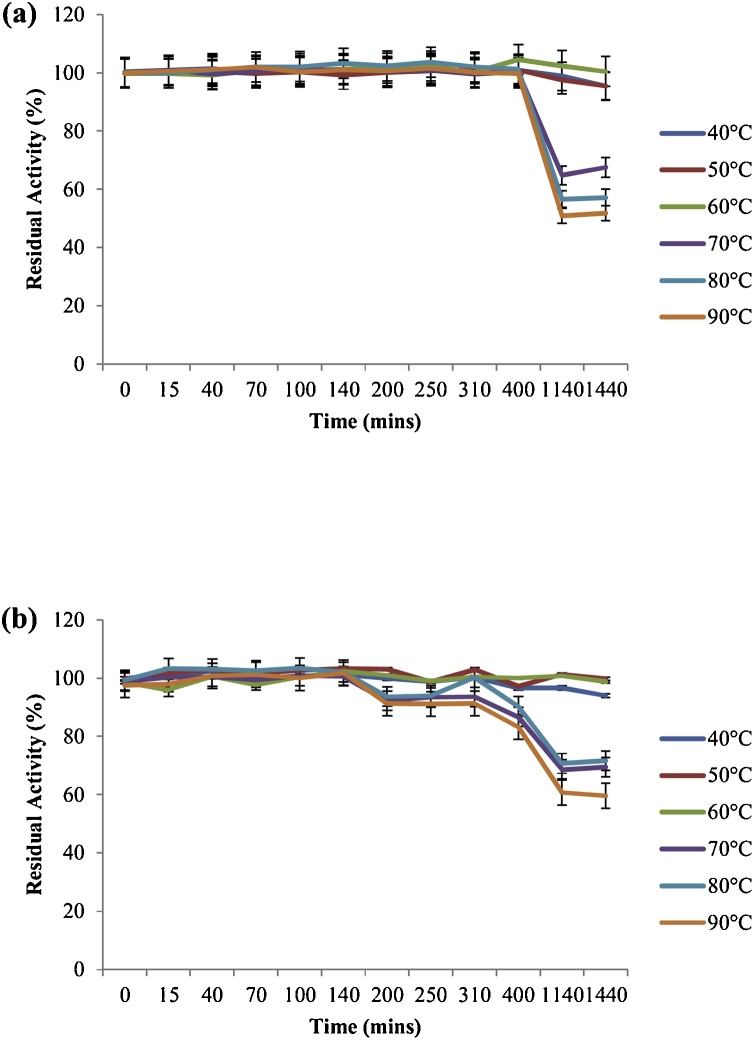
Effect on temperature on the stability of laccases from (a) Hb16c (*Bordetella bronchiseptica* HSO16) and (b) Berl1^1^b_2_ (*Stenotrophomonas maltophilia* BIJ16) with ABTS as substrate.

**Fig. 3:**
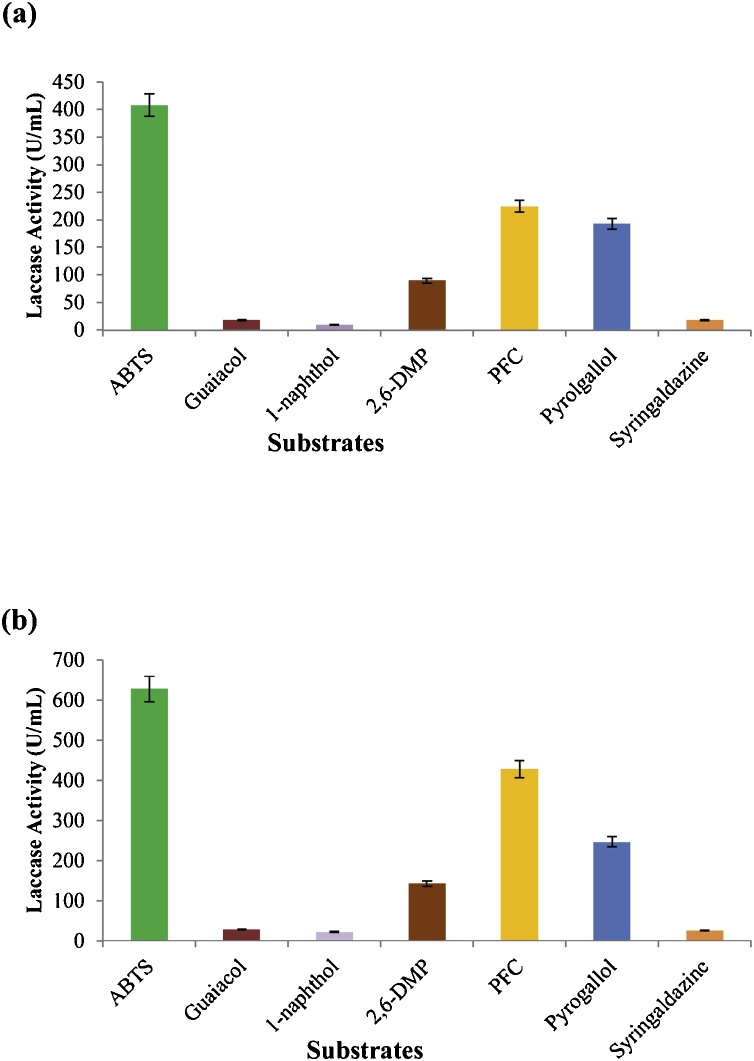
Pattern of substrate specificity in (a) *Bordetella bronchseptica* HSO16 and (b) *Stenotrophomonas maltophilia* BIJ16.

Some unique biochemical properties of the laccase secretions appraised in this study have been outlined in Table 2a, Table 2b. Both laccases had pH optima of 6 (Hb16c) and 7 (Berl1^1^b_2_) respectively, whereas relative activity ranged from ca. 67 to 97% for Hb16c and ca. 86–95% for Berl1^1^b_2_, in the pH regimes 3–11. This robustness in laccase activity has been earlier reported [4], where about 85% relative activities were observed for *Citrobacter* sp. at pH 3 and 11 respectively. Similarly, a purified laccase from *Trametes pubescens*, with pH 5 optima, displayed at least 80% relative activity from pH 5 till pH 11 [8]. However, an acidophilic laccase with pH 4 optima [9] and an alkalophilic laccase with pH 9 optima [10] have been identified. Interestingly, the enzymes from both bacterial strains had an enhanced stability for over 455 min in buffered aqueous systems ranging from pH 3 to 11 (Fig. 1), with minimum residual activity estimates of 99% (Hb16c) and 100% (Berl1^1^b_2_) respectively. Although the exact reason for this outcome cannot be proffered at the moment, the author ratiocinates that the robustness of activity might be attributed to the heterogeneity of cofactors in the catalytic center of the laccases, since diversity in cofactor composition might elicit distinct properties characteristic of the enzyme-cofactor interactions. This outcome is corroborated by an earlier report, which exhibited absolute versatile pH stability of the laccases assessed [4]. Das et al. [11] reported remarkable stabilities in pH 3 and 6.8 solutions, but a different assay substrate was employed in their investigation. Moreover, at least 85% residual activities were recovered from the laccases after refrigeration (4 °C) for about 23 weeks in buffers ranging from pH 3–11 (data not shown), thereby suggesting their prospects for long-term preservation. Optimal temperatures were observed at 60 °C (Hb16c) and 70 °C (Berl1^1^b_2_); even so, remarkable relative activities were recorded at 0 °C (Hb16c: ca. 71%; Berl1^1^b_2_: ca. 81%) and 90 °C (Hb16c: ca. 83%; Berl1^1^b_2_: ca. 87%) respectively. Comparably high optimal temperatures have been recorded by several authors [[12], [13], [14]]. Stability studies presented a trend of robustness at the temperature profiles assessed (40 °C – 90 °C), for over 400 min (Fig. 2). However, incidental inspections at 1140 and 1440 min revealed tremendous residual activities, especially at temperatures ranging from 40 °C to 60 °C (Hb16c: ca. 95–100%; Berl1^1^b_2_: ca. 94–100%). Rezaei et al. [15] reported a 6 h stability (ca. 80% residual activity) at 25−55 °C; however at 75 °C, only about 50% of its activity was measurable. Both laccases were favourably responsive to different concentrations of cations and surfactants (Table 2a); Hb16c had a reduced activity at 2.5 mM Cu^2+^ (ca. 92%), whereas Berl1^1^b_2_ expressed maximum activity, when incubated with 5 mM Cu^2+^ (ca. 125 %). Even so, Hb16c was better induced by all assessed concentrations of Mg^2+^, Co^2+^ and Zn^2+^ than Berl1^1^b_2_. Furthermore, both laccases had their activities enhanced (ca. 103% – ca. 120%) on incubation with surfactants (EDTA, Benzoic Acid, SDS), except Berl1^1^b_2_, which had a residual activity of ca. 97%, when incubated with 5 mM benzoic acid. The chloride inducement of laccase activity of observed in both isolates, even at novel high concentrations; Hb16c recorded ca. 108% residual activity in 40% NaCl, while ca. 123% was observed for Berl1^1^b_2_. Conversely, a reversal of activities was observed for both secretions, when incubated in NaF, the most electronegative halide (Table 2b). Despite the renowned inhibitory effects of NaF [16], Hb16c maintained its inducement response, presenting residual activities ranging from ca. 104% to ca. 109%, when exposed to 2%, 5%, 10% and 20% NaF, respectively, whereas only 5% NaF could induce an experiential increase in residual activity in Berl1^1^b_2_ (ca. 105%); albeit other concentrations could still afford outputs ranging from ca. 92% to 93%. Among the solvents tested, Tween 20 elicited greater residual activities, overall, when preincubated with the two laccases (Table 2b). Overall, the unique metal-, halo- and surfacto-tolerance displayed by these laccase secretions have only been reported by Unuofin et al. [4]; albeit few authors have been able to report decent levels of tolerance in their studies [9,14,[17], [18], [19]]. Moreover, the astounding range of residual activities observed at high concentrations of solvents (20% and 40%) suggest their applicability in reaction conditions that require the dissolution of substrates on which they would oxidize. It was subsequently observed that Hb16c and Berl1^1^b_2_ shared similar patterns of substrate specificity, in the sequence ABTS > PFC > pyrogallol > 2,6–DMP > guaiacol > syringaldazine > 1-napththol (Fig. 3). Generally, the synthetic non-phenolic substrates (ABTS and PFC) were more rapidly oxidized compared to their phenolic *confrère*. This observation was earlier reported by Unuofin et al. [4], and it might be due to their high redox potentials, which could stimulate the rapid generation of radicals for their reaction cascades; this observation might be useful in treatment of majority of wastewater pollutants, which are of synthetic orientation. ABTS was subsequently adopted for kinetic studies (Table 3), which generated values extrapolated from Lineweaver-Burk plots with regression coefficients of 97.7 (Hb16c) and 96.6 (Berl1^1^b_2_). A tremendous kinetic profile was observed in Berl1^1^b_2_ as compared to Hb16c; it exhibited a higher specificity (*K_cat_*/*K_m_*: 1.2 × 104 μM^−1^ S^−1^), substrate affinity (*K_m_*: 0.636 μM) and catalytic constant (*K_cat_*: 7.57 × 10^3^ S^−1^). Tremendous energetics has been reported from secretions of *Achromobacter xylosoxidans* and *Citrobacter freundii* respectively [4]; whereas a member of the firmicutes was reported to possess enhanced energetics, particularly its affinity for ABTS [15]. Be that as it may, both secretions could still be ratified as prolific biocatalysts, based on their individual kinetic properties, for overarching applications in industrial biotechnology.

**Table 2a:** Effect of pH regimes, metalsand surfactants on Hb16c and Berl1^1^b_2_ laccase activity.

	Relative Activity (%)				Residual Activity (%)	
pH	Hb16c	Berl1^1^b_2_	Metals and Surfactants	Concentration (mM)	Hb16c	Berl1^1^b_2_
3	67.48±1.06^a^	85.97±1.14^a^	None	_	100	100
4	78.18±1.28^b^	88.83±1.02^b^	Mg^2+^	1	110.62 ± 1.63^a^	97.19 ± 4.24^a^
5	91.78±1.16^c^	94.73±1.32^d^		2.5	108.88 ± 1.22^a^	95.75 ± 3.92^a^
6	100	93.24±0.95^d^		5	108.69 ± 3.04^a^	94.41 ± 3.66
7	97.06±1.27^e^	100		7.5	110.87 ± 2.44^a^	94.43 ± 3.42^a^
8	93.04±1.54^d^	90.03±1.64^c^	Co^2+^	1	111.18 ± 1.18^a^	106.47 ± 1.38^d^
9	90.72±2.21^c^	93.89±1.48^d^		2.5	112.03 ± 1.64^a^	100.71 ± 2.36^a^
10	90.38±1.02^c^	88.25±1.24^b^		5	112.22 ± 3.58^b^	101.96 ± 2.16^a^
11	90.16±2.03^c^	85.44±1.37^a^		7.5	112.72 ± 2.08^a^	105.02 ± 1.04^d^
			Zn^2+^	1	109.27 ± 2.32^a^	93.04 ± 3.14^c^
				2.5	110.27 ± 1.88^a^	90.39 ± 4.04^b^
				5	113.59 ± 3.22^b^	88.65 ± 3.66^a^
				7.5	111.20 ± 4.02^a^	98.79 ± 2.22^d^
			Cu^2+^	1	110.34 ± 1.74^e^	111.31 ± 4.86^c^
				2.5	92.38 ± 1.52^a^	97.04 ± 2.15^a^
				5	98.29 ± 2.16^b^	124.92 ± 0.86^e^
				7.5	103.36 ± 1.72^c^	119.24 ± 1.13^e^
			EDTA	1	113.09 ±0.92^b^	111.62 ±1.16^a^
				2.5	115.85 ±1.25^c^	110.16 ±1.44^a^
				5	112.03 ±1.66^b^	114.10 ±1.3^b^
				7.5	109.44 ±1.32^a^	109.35 ±1.48^a^
			Benzoic Acid	1	115.48 ±1.58^a^	109.75 ±1.28^c^
				2.5	117.81 ±2.28^b^	103.45 ±1.27^b^
				5	116.25 ±1.74^b^	96.98 ±1.32^a^
				7.5	113.04 ±1.66^a^	107.17 ±1.66^c^
			SDS	1	119.75 ±1.25^b^	111.05 ±1.08^b^
				2.5	114.72 ±2.85^a^	106.13 ±1.92^a^
				5	115.88 ±1.78^a^	110.96 ±1.23^b^
				7.5	117.87 ±2.36^b^	111.41 ±1.37^b^

Values represent mean ± SD of three replicates.Values with the same superscript alphabet along the same column segment are not significantly different (*P > 0.05*).

**Table 2b:** Influence of temperature, halides and solvents on Hb16c and Berl1^1^b_2_ laccase activity.

	Relative Activity (%)				Residual Activity (%)	
Temperature (°C)	Hb16c	Berl1^1^b_2_	Halides and Solvents	Concentration (%w v^−1^)	Hb16c	Berl1^1^b_2_
0	71.37 ± 1.06^a^	81.46 ± 1.14^a^	None	_	100	100
20	82.40 ± 1.28^b^	84.76 ± 1.06^a^	NaCl	5	101.55 ± 1.63^a^	114.05 ± 4.24^a^
30	91.88 ± 1.22^c^	90.56 ± 1.32^b^		10	114.05 ± 1.22^d^	118.03 ± 3.92^b^
40	90.19 ± 1.18^c^	91.88 ± 0.95^c^		20	111.64 ± 3.04^d^	116.21 ± 3.66^b^
50	95.90 ± 1.27^d^	91.65 ± 1.04^c^		40	108.09 ± 2.44^c^	123.52 ± 3.42^e^
60	100	96.90 ± 1.52^d^	NaF	2	109.56 ± 1.18^b^	93.46 ± 1.38^a^
70	97.16 ± 1.15^d^	100		5	105.74 ± 1.64^a^	105.32 ± 2.36^c^
80	94.97 ± 1.2^c^	91.50 ± 1.26^b^		10	106.15 ± 3.58^a^	92.14 ± 2.16^a^
90	83.48 ± 1.08^b^	86.86 ± 1.16^a^		20	104.33 ± 2.08^a^	92.39 ± 1.04^a^
			DMSO	5	107.56 ± 2.32^c^	106.37 ± 3.14^c^
				10	109.77 ± 1.88^d^	100.43 ± 4.04^a^
				20	91.57 ± 3.22^a^	101.72 ± 3.66^a^
				40	93.19 ± 4.02^a^	102.03 ± 2.22^a^
			Tween 20	5	100.71 ± 1.74^b^	119.37 ± 4.86^d^
				10	87.40 ± 1.52^a^	102.46 ± 2.15^a^
				20	114.14 ± 2.16^d^	105.24 ± 0.86^a^
				40	108.19 ± 1.72^c^	114.54 ± 1.13^c^

Values represent mean ± SD of three replicates. Values with the same superscript alphabet along the same column are not significantly different (*P > 0.05*).

**Table 3:** Kinetic characteristics of bacterial laccases.

Laccase type	*V_max_* (*μM min^−1^* mg^-1^)	*K_m_* (*μ*M)	*K_cat_* (s^−1^)	*K_cat_/K_m_* (*μ*M^−1^ s^−1^)
*Bordetella* sp. HSO16 laccase	303.03	1.090	5.05 × 10^3^	4.6 × 10^3^
*Stenotrophomonas* sp. BIJ16 laccase	454.54	0.636	7.57 × 10^3^	1.2 × 10^4^

### 3.2 Laccase-encoding genes

**Fig. 4:**
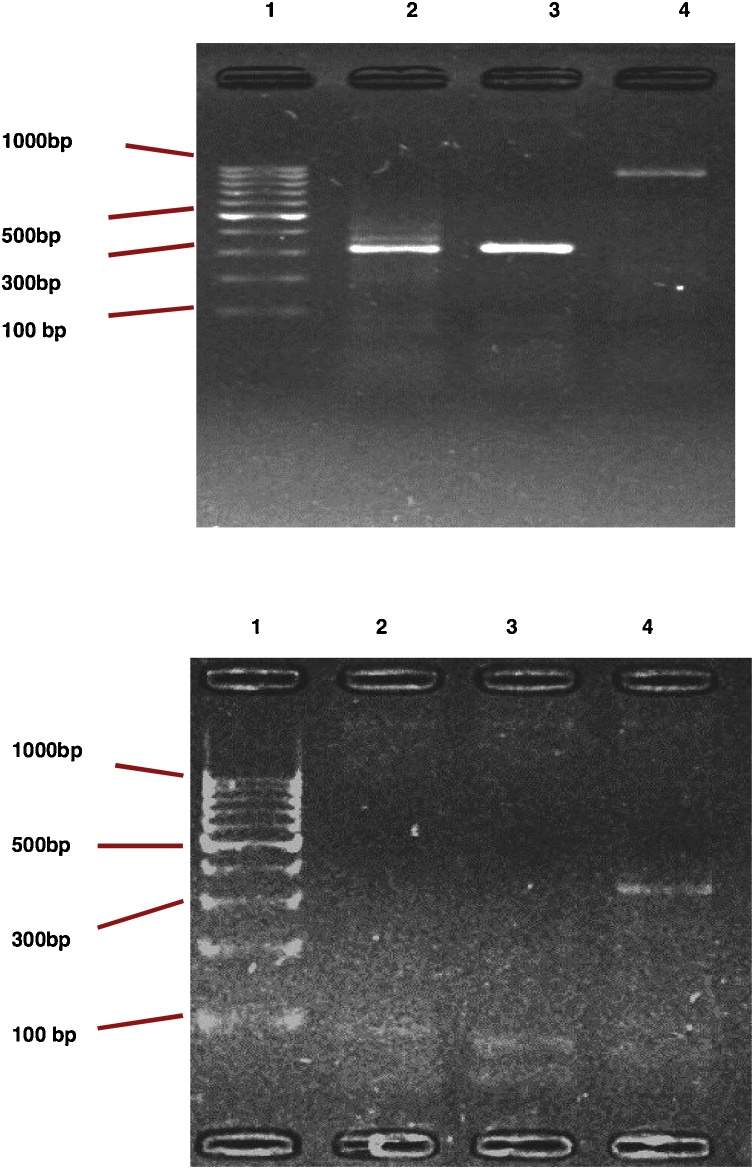
Molecular snapshot of amplified fragments of laccase genes from (a) *Bordetella bronchiseptica* HSO16 and (b) *Stenotrophomonas maltophilia* BIJ16. Lane 1: ladder mix, lane 2: CueOP gene, lane 3: MCOStm gene, lane 4: CueOCit gene.

In this study, the presence of multiple homologous laccase-encoding genes was inferred from a snapshot molecular analysis (Fig. 4). It was ratiocinated as the possible reason for the robust biochemical novelty of the assessed laccase secretions, since the expression of each gene might be responsible for the particular function of the isozyme in the secretion. This outcome had been earlier reported by Unuofin et al. [4], where at least five distinguishable homologous gene sequences were located through gel electrophoresis. Although fungi were the first, and are the most extensively studied for occurrences of multiple laccase genes [20,21], the bacterial laccase-like genes might be more participatory in laccase activity than their fungal *confrère*. Moreover, the absence of a glycosylated carbohydrate fraction, which is the extant constraint for genetic manoeuvrability and heterologous expression in fungi, makes bacteria most suitable for genetically engineered process and applications pertinent to the overwhelming demands of our present society [22].

### 3.3 Laccase decolorization of synthetic dyes

**Fig. 5:**
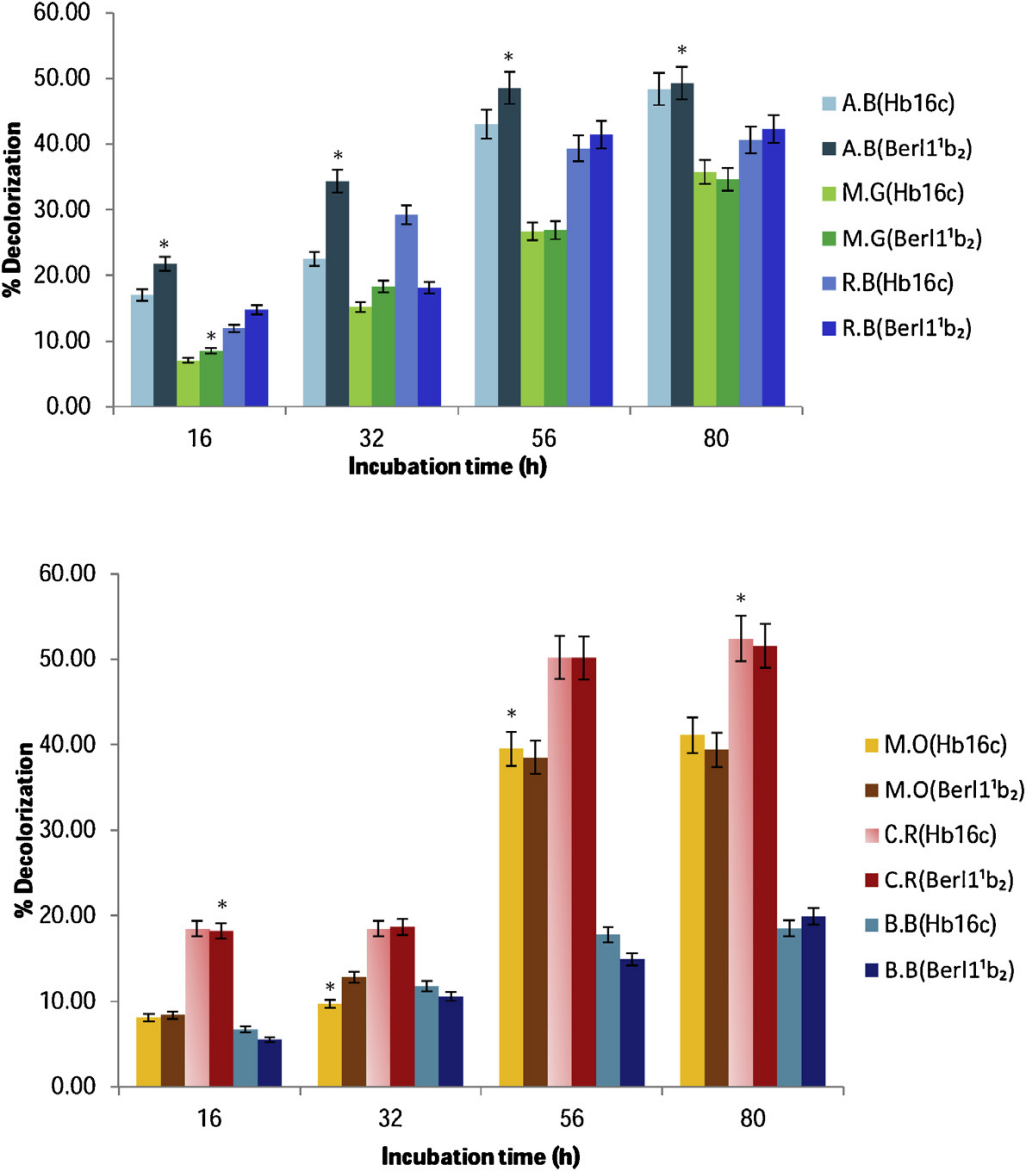
Decolourization of different classes of synthetic dyes (0.2 %) by Hb16c (*Bordetella bronchiseptica* HSO16) laccase, and Berl1^1^b_2_ (*Stenotrophomonas maltophila* BIJ16) laccase. A.B; azure B, M.G; malachite green, R.B; reactive blue, M.O; methyl orange, C.R; congo red, B.B; brilliant blue, Imm; immobilized laccase. Phosphate buffer (pH 6) was used and no mediator was added. Error bars are deviations from means of replicate readings; asterisks indicate significant difference (*P < 0.05*) among treatments (For interpretation of the references to colour in this figure legend, the reader is referred to the web version of this article).

The biotechnological potential of the secretions was appraised in the degradation of synthetic dyes of the heterocyclic cationic, triarylmethane, azo and anthraquinone groups, at novel high concentrations (0.2% w v^−1^), without redox mediators. From the degradation profile portrayed in Fig. 5, a linear decolourization pattern was observed for both secretions (Hb16c and Berl1^1^b_2_) in AB, MG and RB aqueous matrices; however, a marginal increase was observed for Berl1^1^b_2_ treated AB medium between 56 h and 80 h incubation period. Similarly, a phenomenal decolourization occurred between 32 h and 56 h of incubation, when laccase secretions were incubated with MO and CR; however, no significant increase was observed on terminal incubation (80 h). Maximum decolourization was observed in CR on terminal incubation (Hb16c: ca. 52.5% and Berl1^1^b_2_: ca. 51.6%), whereas minimum decolourization was portrayed by BB, where only about 20% decolourization was realized. To my knowledge, this might be the highest dye concentration assessed on a laboratory scale; hence the low levels of decolourization among some of the dyes assessed. Moreover, steric hinderances that might be caused by the atomic orientation of the dyes could make laccase accessibility relatively unachievable. However, these dyes are getting increasingly susceptible to laccase catalysis [23,24]. Therefore, further work is anticipated to enhance the biodegradation capacities of these isolates, as they may be germane to diverse environmental applications. Although, to my knowledge, reports regarding decolourization of the concentrations of dyes employed in the present investigation are particularly scarce, the studies of the selected authors would be deemed insightful [[25], [26], [27], [28]].

### 3.4 Denim bioscouring

**Fig. 6:**
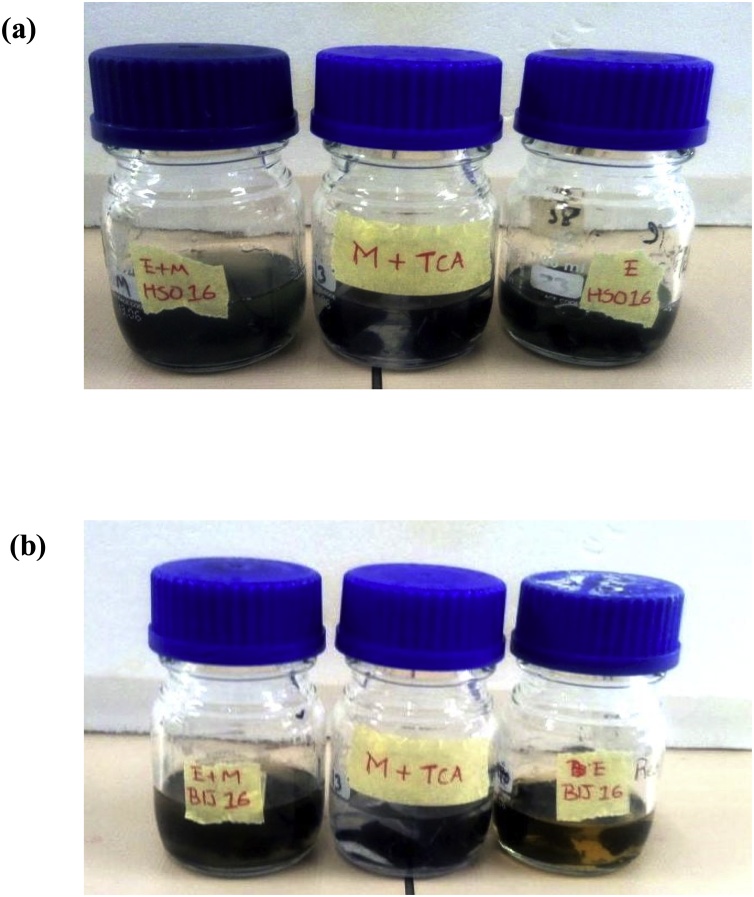
Empirical denim bleaching using; (a) Hb16c (*Bordetella bronchiseptica* HSO16) laccase, and (b) Berl1^1^b_2_ (*Stenotrophomonas maltophilia* BIJ16) laccase. Enzyme + Mediator (E + M) is on the left; Enzyme only treatment (E) is on the right, while the middle (M + TCA) is the control containing the mediator and TCA.

**Fig. 7:**
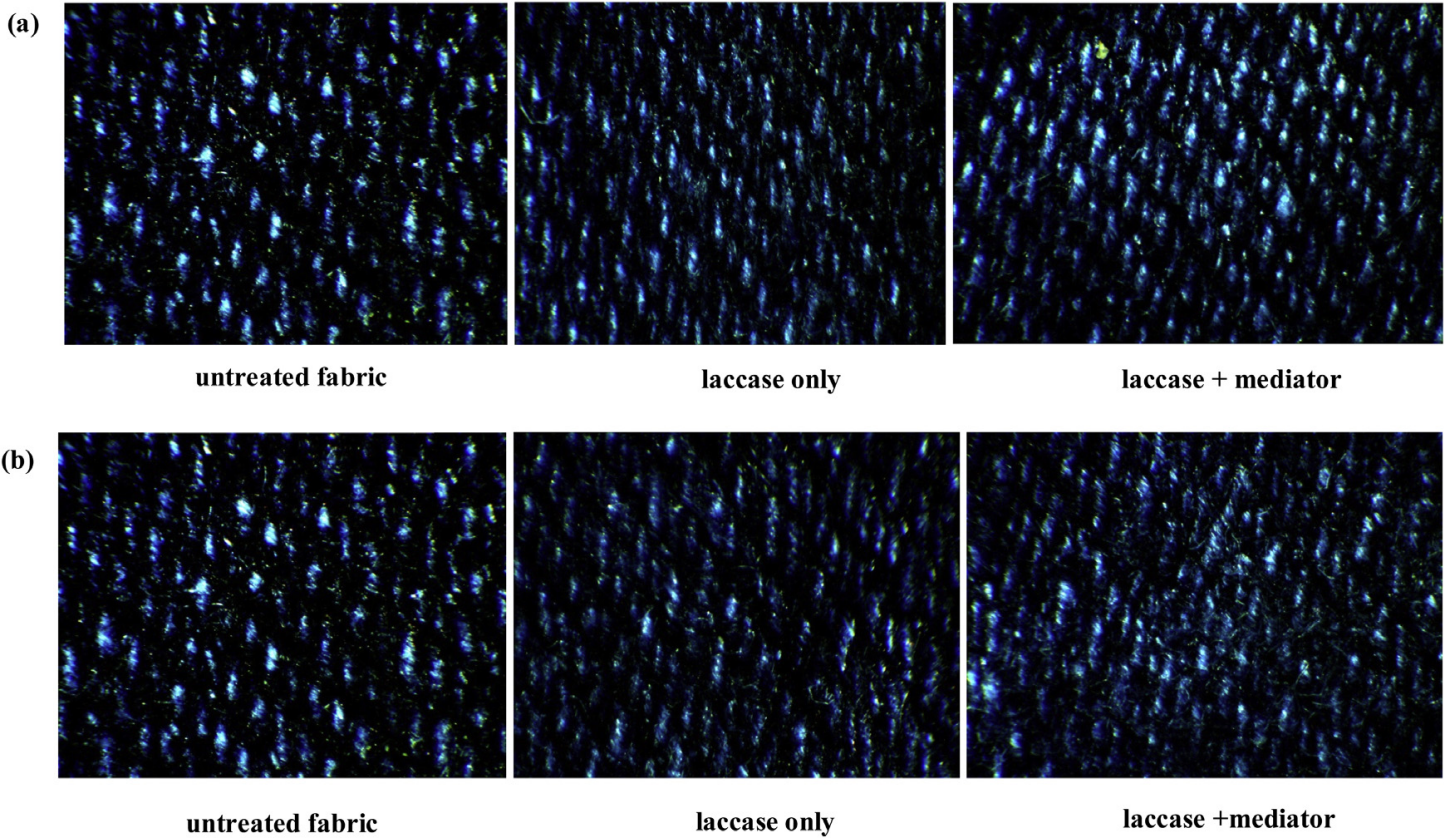
× 30 magnification of the dissection microscope: Denim bioscouring by (a) Hb16c (*Bordetella bronchiseptica* HSO16) and (b) Ie1c (*Stenotrophomonas maltophilia* BIJ16). Comparisons were made between untreated fabric (control) and the treated variants; laccase only (middle), and laccase + mediator; ABTS (right).

**Fig. 8:**
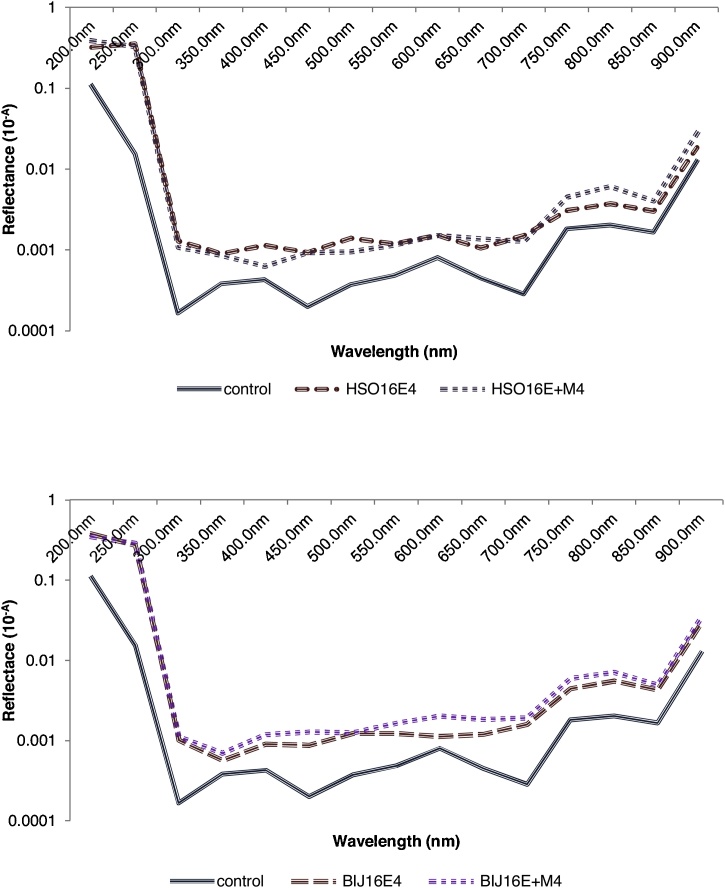
Spectrum plots of denim treated with laccase, and ABTS + laccase from, (a) *Bordetella bronchiseptica* HSO16, (b) *Stenotrophomonas maltophilia* BIJ16. Absorbance (A) of samples was read from 200 to 900 nm and the log inverse (10^−A^) was plotted as reflectance.

No immediate experiential leaching of pigments into the surrounding medium was observed when only laccase tinctures were added. However, an amalgam comprising laccase tinctures and a mediator (ABTS) into the medium (E + M), elicited a drastic change of the medium to green, after the addition of TCA – a component of the laccase assay, which is iconic of laccase oxidation of ABTS. Briefly thereafter, the colour of the medium was changed to the denim’s pigment, indicating the emprical leaching of denim dye into the surrounding medium (Fig. 6). A control, which had all other components of the reaction mixture, except laccase, which was replaced with buffer, was set up in order to ascertain the anticipatable effect of TCA on the denim, since it is widely recognized as a potential bleaching agent. From the results observed, it could be suggested that the bioscouring activity of the laccases was enhanced only when the amalgams were oxidized; however, this phenomenon has been satisfactorily elucidated by only a few investigators [29]. It was observed that the fabrics had a brighter hue on modification with enzyme treatment, however, in the presence of the mediator, fabric hue was brightest (Fig. 7). This outcome has been corroborated through colorimetric assessment [29], though the laccase individually-treated fabric assessed by the aforesaid investigators did not show any glaring difference from the untreated, just as was observed in this study. This implies that the laccases investigated, only needed the oxidized mediators for acceleration of the bleaching process. Conversely, a different mediator, violuric acid, had been used to achieve a similar outcome to the one observed in this study [30]; another mediator that has been successfully applied is 1-hydroxybenzotriazol (HBT) [31]. Recently, a purified manganese peroxidase extract from *Cerrena unicolor* was capable of enhanced denim bleaching without the exogenous supply of mediators [32]. However, the potential role of hydrogen peroxide (H_2_O_2_) in aiding bleaching should be scrutinized, since it was applied as a cosubstrate during treatment. Other historian approaches to denim bleaching include the collaborative investigations of Maryan and Montazer [[33], [34], [35]], and the essential discovery of Pazarlioğlu et al. [36]. A recent notable mention is the study of Singh et al. [37], which highlighted the acetosyringone-mediated laccase complete degradation of indigo carmine (50μM) over 10 min of incubation, though its evaluation on indigo-affixed denim was not reported. Consequently, other oxygenases might possess the denim bioscouring ability; this has been confirmed in a recent study [32]. From Fig. 8, the constrast between the untreated and the treated is comparable to what was reported by Iracheta-Cárdenas et al. [30], where the treated fabric recorded a higher reflectance reading. Ultimately, a striking phenomenon, which was observed in a similar study (Unuofin et al. unpublished), is the step by step decolourization of the dye bath, hours after the initiation of scouring. A precise explanation for this phenomenon is not identifiable at present, but efforts are being made to understand the mechanism of this dual reaction (denim bleaching and dye decolourization) in order to improve thereupon and create a seamless dyeing of denims and the concomitant cleaning of dye baths. Moreover, the step by step decolourization should be monitored for spinoffs, which might serve as valuable startup chemicals for various industrials applications. Given these astronomical qualities of the tinctures, an environomical approach proposed by Unuofin et al. [22] should be adopted to enable the simultaneous bulk production of these fine biochemicals as well as the contribution of spent biomass to sustainable agriculture, which will, on the whole, promote environmental sustainability.

## 4 Conclusions

In this study, the journey to sustainability began with two bacteria isolates (Hb16c and Berl1^1^b_2_), which were isolated from environmental wastes and further utilized the wastes as feedstock for the production of a fine biochemical, laccase. These tinctures were shown to have novel biochemical properties bespoke for a wide range of environmental applications, such as the decolourization of a novel high concentration of synthetic dyes, as well as the corresponding bleaching of denim and effluent decolourization all at once. The outcomes of this investigation therefore propose a blueprint for sustainability, since they highlight the essence of environmental mining of strategic bacteria with immense physiological and biochemical machinery for the abatement of pollution, and the conversion of waste to bioresources.

## Declaration Competing Interest

None.
